# E2F1 Drives Breast Cancer Metastasis by Regulating the Target Gene FGF13 and Altering Cell Migration

**DOI:** 10.1038/s41598-019-47218-0

**Published:** 2019-07-24

**Authors:** Daniel P. Hollern, Matthew R. Swiatnicki, Jonathan P. Rennhack, Sean A. Misek, Brooke C. Matson, Andrew McAuliff, Kathleen A. Gallo, Kathleen M. Caron, Eran R. Andrechek

**Affiliations:** 1 Lineberger Comprehensive Cancer Center University of North Carolina, Chapel Hill, United States; 2 Department of Physiology, Michigan State University, East Lansing, United States; 3 University of North Carolina Department of Cell Biology, Chapel Hill, United States

**Keywords:** Metastasis, Tumour immunology

## Abstract

In prior work we demonstrated that loss of E2F transcription factors inhibits metastasis. Here we address the mechanisms for this phenotype and identify the E2F regulated genes that coordinate tumor cell metastasis. Transcriptomic profiling of E2F1 knockout tumors identified a role for E2F1 as a master regulator of a suite of pro-metastatic genes, but also uncovered E2F1 target genes with an unknown role in pulmonary metastasis. High expression of one of these genes, Fgf13, is associated with early human breast cancer metastasis in a clinical dataset. Together these data led to the hypothesis that Fgf13 is critical for breast cancer metastasis, and that upregulation of Fgf13 may partially explain how E2F1 promotes breast cancer metastasis. To test this hypothesis we ablated Fgf13 via CRISPR. Deletion of Fgf13 in a MMTV-PyMT breast cancer cell line reduces colonization of the lungs in a tail vein injection. In addition, loss of Fgf13 reduced *in vitro* cell migration, suggesting that Fgf13 may be critical for tumor cells to escape the primary tumor and to colonize the distal sites. The significance of this work is twofold: we have both uncovered genomic features by which E2F1 regulates metastasis and we have identified new pro-metastatic functions for the E2F1 target gene Fgf13.

## Introduction

Breast cancer progression to metastatic disease is associated with poor prognosis, with only 22% of the patients surviving five years^1^. As a result, there is a critical need to understand the molecular mechanisms that regulate metastasis. High throughput transcriptomic assays have been pivotal in understanding alterations in the transcriptional programs of cancer cells during the various steps of metastasis. Repeated selection of cells with the propensity for organ-specific metastasis^2–4^ led to the discovery of transcriptional signatures unique to each metastatic site with characteristic transcriptomic changes^5^. Other studies have combined clinical observations with gene expression profiling to generate gene signatures that predict progression to metastasis^6–8^. Finally, gene expression profiling has been used to identify genes involved in metastasis which were then validated in genetically engineered mouse models of breast cancer^9–12^.

One of the best characterized models of breast cancer metastasis is the MMTV-PyMT system where the expression of the polyoma virus middle T antigen is expressed under the control of the mouse mammary tumor virus promoter/enhancer^13^. Expression of the middle T antigen results in activation of key signaling pathways including Ras, PI3K/AKT and PLC-γ. These transgenic mice rapidly develop multifocal mammary tumors and develop pulmonary metastasis at endpoint with nearly 100% penetrance. Our recent work highlighted the similarities of MMTV-PyMT tumors to human breast cancer and identified shared gene expression alterations between this mouse model and human disease^14^. One example is our observation that E2F pathway signatures were elevated in the MMTV-PyMT model, and we ultimately validated the role for E2F1, E2F3, and E2F3 in tumor progression using mouse models^11,14^. E2F transcription factors are canonically involved in the G1/S transition, ultimately either promoting (E2F1-3a) or suppressing (E2F3b-8) cell cycle progression^15–17^. In this study we expand upon this theme to demonstrate a role for the activator class of E2Fs in tumor progression independent of their role in cell cycle progression.

Interbreeding MMTV-PyMT mice with mice null for E2F1^18^, E2F2^19^ and E2F3^20^ resulted in alterations in mammary gland development, tumor latency, histology, and vascularization^11,21^. In addition to, or perhaps as an effect of, the role of E2F1 and E2F2 on these tumor phenotypes, E2F1 and E2F2 deletion reduced metastatic capacity accompanied by a decrease in circulating tumor cells. This led to the hypothesis that the E2F transcription factors regulate tumor cell intrinsic gene expression programs that are critical for metastatic progression.

Given that E2F transcription factors have been demonstrated to bind thousands of individual target genes^22^, we sought to characterize the gene expression profiles of E2F1^−/−^ MMTV-PyMT tumors. We hypothesized that this approach would allow us to identify E2F1-mediated transcriptional programs which contribute to metastasis. This would allow us to identify E2F1 target genes which were previously implicated in breast cancer metastasis but would also allow to identify novel metastasis driver genes. In line with this, we have identified a suite of E2F1 target genes which have been previously implicated in breast cancer metastasis, suggesting that E2F1 may be a master regulator of breast cancer metastasis. In addition, this analysis has also identified a new role for the E2F1 target gene fibroblast growth factor 13 (Fgf13) in breast cancer metastasis.

## Results

### Genomic comparison of E2F^WT/WT^ and E2F^−/−^ tumors

**Figure 1:**
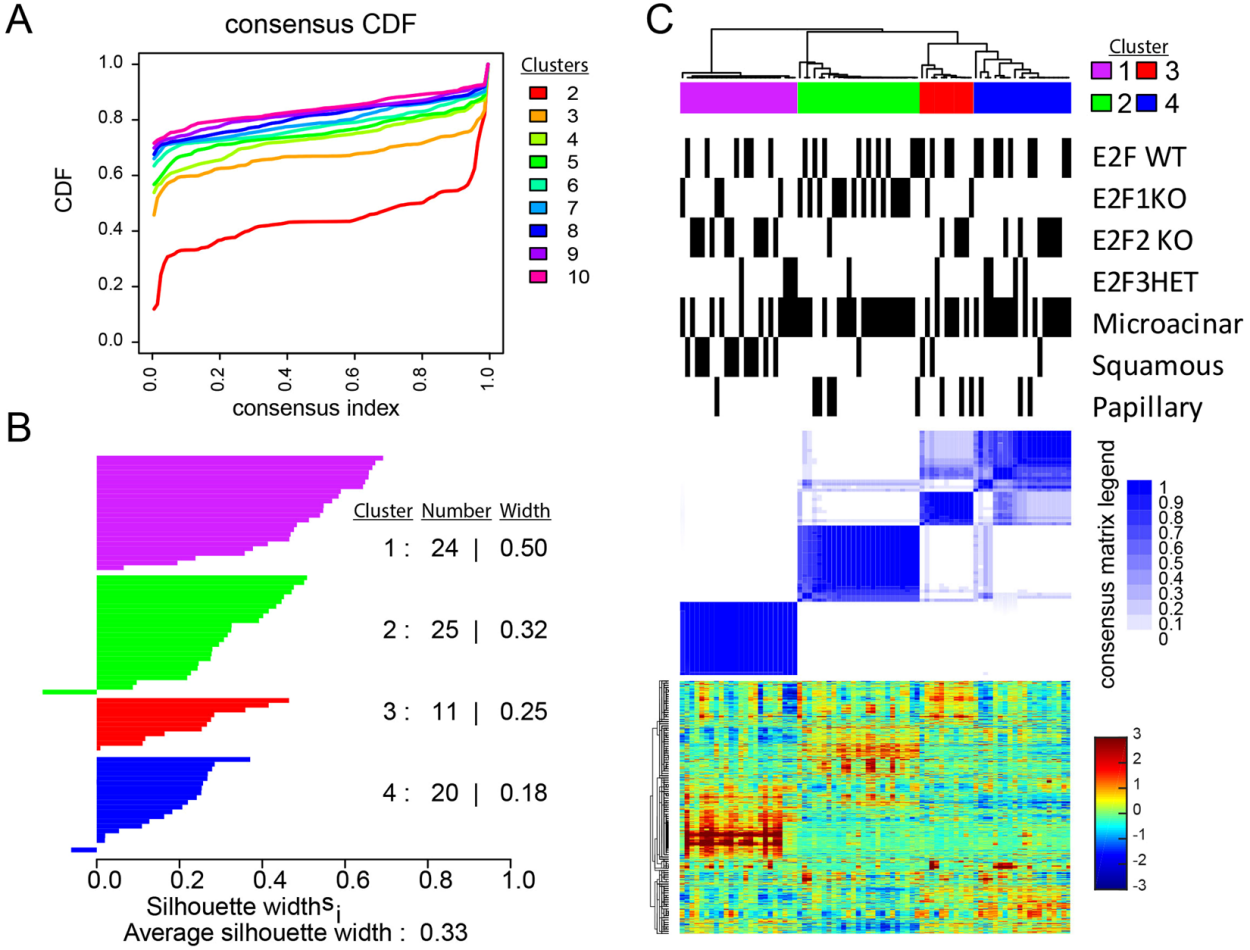
Consensus clustering reveals the major gene expression classes of MMTV-PyMT E2F knockout tumors. (**A**) Unsupervised class discovery of MMTV-PyMT tumor gene expression data (GSE 104397) by measure of empirical cumulative distribution functions^23^. The x-axis displays the consensus index, which is a measure of samples clustering together (0.0 samples that never cluster together, 1.0 samples that always cluster together). On the y-axis the CDF value is displayed. This provides a measure of cluster stability. Thus, this plot measures which number of clusters (as color coded) that provide maximum CDF (stable classification) for samples that are ambiguous (may not always cluster together). (**B**) A silhouette width^24^ analysis based upon the four clusters identified measures the correlation of samples within each cluster (the higher the silhouette width, the higher the correlation of a given sample to other samples in the cluster; each bar represent a sample). (**C**) The consensus cluster based upon the four selected clusters (as determined in **A**). Consensus clustering was performed using 1,000 iterations and 90% item (sample) resampling. The dendrogram across the top shows the relationship of saples on the basis of gene expression profiles. Below the dendrogram, color coded boxes itemize the cluster labels and concordant to those shown in panel B. Next, black bars provide sample annotations for genotype and tumor histology according to sample position in the dendrogram above and heatmaps below. Next, the blue heatmap illustrates the sample and cluster relationships over the 1,000 iterations of 90% resampling and cluster classification. The darkest blue indicates samples that co-clustered 100% of the time, white indicates samples that never clustered together. For samples with intermediate values, the percentage of co-clustering with other samples outside the cluster is shown. For example, the far left samples in cluster 2, sometimes clustered with samples in cluster 3 over the iterations of resampling a small percentage of the time. Alternatively, cluster 4 samples clustered with cluster 3 samples a small percentage of the iterations resampling and re-clustering. Finally, the heatmap shows the gene expression profiles of each sample (column-wise = samples, row-wise = genes). Genes were ordered using centroid linkage. Expression levels are shown according to the color-bar to the right of the heatmap. (Entire analysis) Data preprocessing: see methods.

To determine the global gene expression response to E2F loss, we analyzed MMTV-PyMT tumors from E2F^WT/WT^, E2F1^−/−^, E2F2^−/−^, and E2F3^+/−^ backgrounds on Affymetrix microarrays. Using an unsupervised classed discovery approach^23^, we investigated the gene expression relationships amongst the various tumors. We used 1000 iterations of data resampling to measure the frequency of co-clustering across 2–10 clusters. Examining potential classes using empirical cumulative distribution functions (CDF) showed that maximum CDF was reached with four clusters (Fig. 1A). This suggests that this cohort of tumors can be divided into 4 distinct clusters, and adding additional clusters will have no statistically significant value^23^. To measure the correlation between samples within each cluster, we used silhouette width^24^ (Fig. 1B). Silhouette width demonstrated the strongest correlations were present in cluster 1. In addition, we observe that the majority of samples have strong similarities to other samples within their assigned cluster. Sample co-clustering across the iterations of resampling is also illustrated in Fig. 1C (see blue-white heatmap, where white shows 0% co-clustering to dark blue 100% clustering). Collectively, this analysis suggests the relationship of these tumors is best described by four clusters.

Examining these clusters in more detail (Fig. 1C), we observe that gene expression patterns correlate with both tumor histology and genotype. For example, cluster 1 (purple) featured mainly tumors with squamous histology. The microacinar and papillary tumors appeared to separate by genotype; with the majority of E2F1^−/−^ tumors ordered into cluster 2, E2F2^−/−^ and E2F3^+/−^ tumors in clusters 3 and 4, and E2F WT tumors were present in each cluster. As an additional analysis, we also used a supervised approach using a published gene set for intrinsic classification of mouse mammary tumors^25^. To adjust for centering biases^26^ and to enable accurate interpretation of this intrinsic analysis, we combined our dataset with published data^9,27,28^ using Bayesian Factor Regression Modelling^29^ (BFRM) to correct batch effects. This intrinsic analysis again separated tumors from the MMTV-PyMT model across distinct clusters (Fig. S1A) and separated tumors according to histology and genotype (Fig. S1B). In agreement with previous work^30^, the squamous tumors showed basal-like gene expression features (Fig. S1B,i). MMTV-PyMT E2F1^−/−^ tumors clustered separately from other MMTV-PyMT tumors and showed high expression of some genes from the luminal cluster (Fig. S1B,ii). As expected, we did not detect evidence for claudin low tumors across any MMTV-PyMT tumors (Fig. S1B,iv). We did not observe differences in the proliferation cluster genes with regards to E2F status (Fig. S1B,iii). However, we did observe a difference by tumor histological subtype. Observing median expression of the proliferation signature genes^31^, microacinar and squamous tumors showed high expression, while papillary tumors had significantly lower median expression (p < 0.01 papillary vs microacinar, or papillary vs squamous; Fig. S2A). In agreement, retrospective analysis of tumor growth rate data, showed that papillary tumors progressed slower (days until 2,000 mm^3^) than microacinar or squamous tumors (Fig. S2B). Taken together, these data demonstrate unique features of tumor histologies and tumor genotypes; with E2F1^−/−^ tumors exhibiting key molecular differences to other MMTV-PyMT tumors.

**Figure 2:**
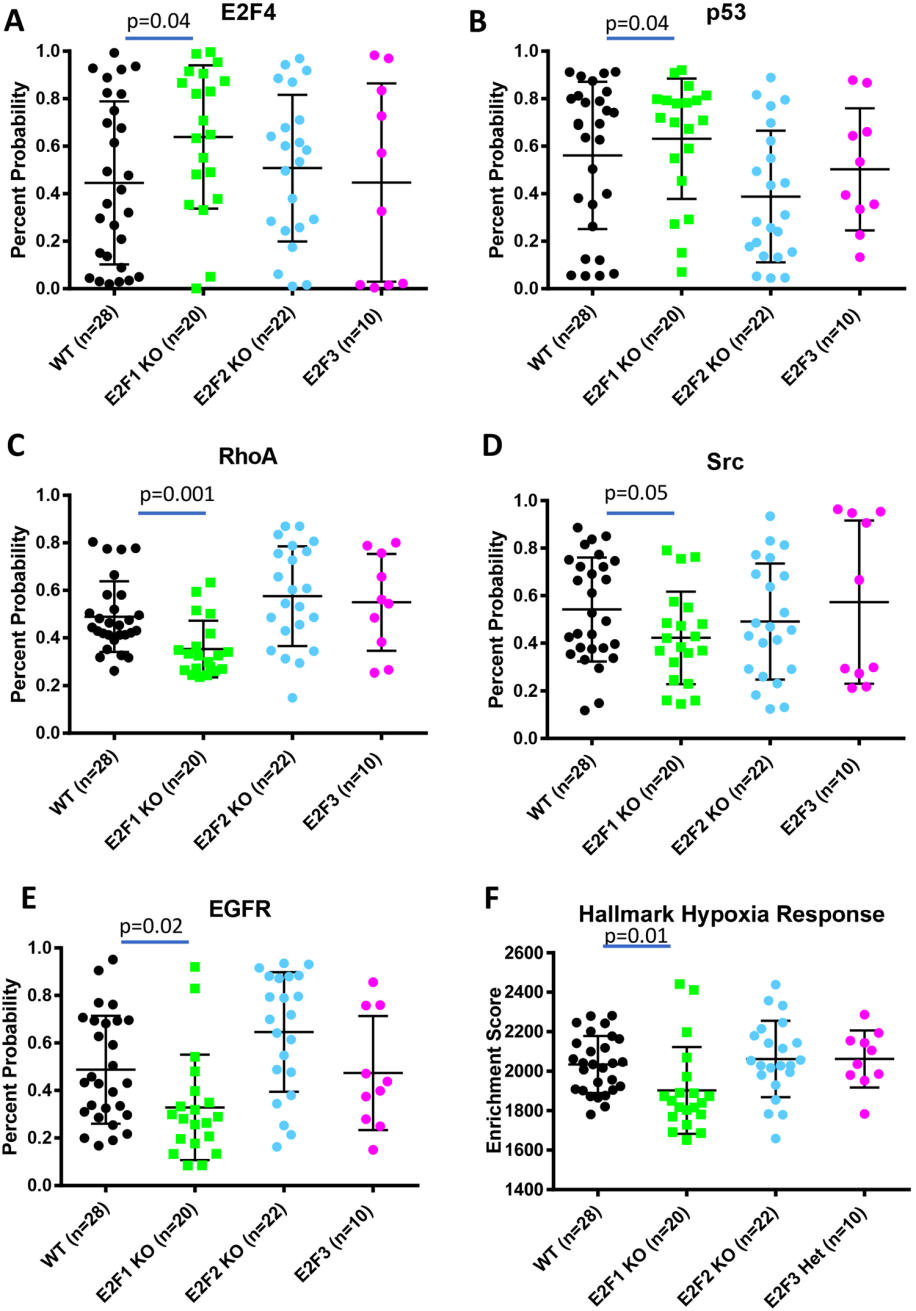
Gene expression signatures reveal pathway activation differences with loss of E2F1. (**A**) Using a binary regression approach^14,32^ to predict E2F4 activity reveals significantly higher probability of E2F4 pathway activation in E2F1 KO tumors compared to E2F WT tumors (p = 0.04). (**B**) Using a binary regression approach^14,32^ to predict p53 activity reveals significantly higher probability of p53 pathway activation in E2F1 KO tumors compared to E2F WT tumors (p = 0.04). (**C**) Using a binary regression approach^14,32^ to predict RhoA activity reveals a significant reduction of probable RhoA pathway activation in E2F1 KO tumors compared to E2F WT tumors (p = 0.001). (**D**) Using a binary regression approach^14,32^ to predict Src activity reveals a reduction of probable Src pathway activation in E2F1 KO tumors compared to E2F WT tumors (p = 0.05). (**E**) Using a binary regression approach^14,32^ to predict Egfr activity reveals a significant reduction of probable Egfr pathway activation in E2F1 KO tumors compared to E2F WT tumors (p = 0.02). (**F**) Using single sample gene set enrichment^36,73^ to analyze expression of the Hallmark Hypoxia Response signature shows a significantly lower enrichment score in E2F1 KO tumors compared to E2F WT tumors (p = 0.01).

To test if these gene expression differences in E2F1^−/−^ tumors corresponded to differences in activation of major cell signaling pathways, we utilized a binary regression approach^32^ to predict pathway activation across the MMTV-PyMT tumors. This revealed E2F1^−/−^ tumors tend to have high activity of E2F4 (Fig. 2A), and p53 (Fig. 2B) pathways and low activity of pathways previously implicated in metastasis; the RhoA^33^, Src^34^, and Egfr^35^ signaling pathways (Fig. 2C–E, respectively). Finally, using single sample gene set enrichment analysis (ssGSEA), we found that E2F1^−/−^ tumors had significantly lower expression of the Hallmark Hypoxia^36^ signature (Fig. 2F); a process also associated with metastasis^37^.

To test for the genes that were significantly differentially regulated between E2F1^−/−^ and E2F^WT/WT^ tumors we used a supervised analysis^38^. Since we hypothesized that E2F1’s role in regulating metastasis was by transcriptional activation of target genes, we were particularly interested in the 226 genes that were significantly downregulated in the E2F1^−/−^ tumors. Many of the genes with low expression in E2F1 KO tumors were associated with hypoxia response (Supplemental File 1).

To begin characterizing these genes for metastatic potential, we used Kaplan Meier analysis of clinically and intrinsically annotated human breast cancer gene expression data^39^. To identify E2F1 target genes, we used ChIPBase^40^. Out of the 226 differentially regulated genes, 98 were E2F1 targets (Supplemental File 1). From these, we focused on genes where high expression in tumors correlated with a decreased time to distant metastasis across all breast cancers as well as within with individual intrinsic subtypes of tumors. We identified 55 genes with pro-metastatic predictions (without discordant predictions in differing subtypes) (Supplemental File 1); 34 of which had demonstrated E2F1 binding sites^22^. Using a Fisher’s exact test, we observed that the distribution of direct E2F1 targets was significantly higher in the genes concordant with human breast cancer metastasis predictions (p = 0.001) than genes either discordant or not predictive of human breast cancer metastasis. This suggested that the E2F1 target genes altered in these tumors are associated with human breast cancer metastatic potential and highlights that E2F1 may be a master regulator of breast cancer metastasis since it controls the expression of multiple pro-metastatic genes.

The 55 genes identified above that were associated with human breast cancer metastasis were examined in the literature to identify which of the molecular changes have already been demonstrated to regulate breast cancer metastasis *in vivo*. As depicted in Fig. S3, Vegfa^41^, Hbegf^42^, Hspb1^43^, Flt1^44^, L1cam^45^, and Plaur^46^ had significantly lower expression (p < 0.05) in E2F1^−/−^ tumors and have all previously been shown to regulate breast cancer metastasis *in vivo*. Additionally, there were genes with significantly lower expression in E2F1^−/−^ tumors that had been shown to have *in vitro* invasion or migration function such as Areg^47^, Tead1^48^, Coro1C^49^, Lama5^50^, Tgm2^51^, and Fgf 7^52^ (p < 0.05, Fig. S4). Taken together, this shows that E2F1^−/−^ tumors have low expression of genes and pathways demonstrated to promote breast cancer metastasis.

### Testing additional genes for metastatic function

**Figure 3:**
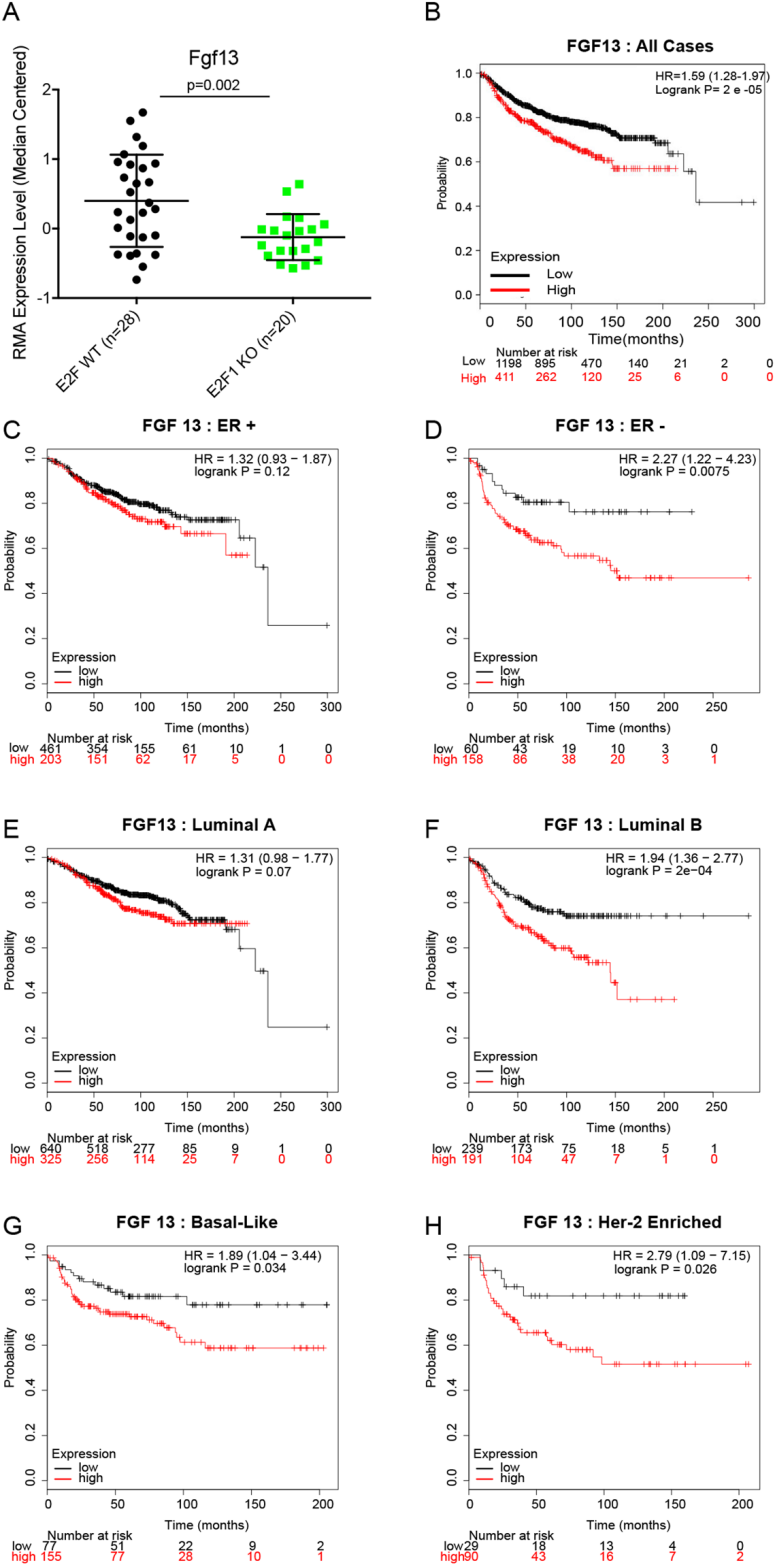
Expression of Fgf 13 is in E2F1 knockout tumors and association with human breast cancer metastasis. (**A**) Boxplot of RMA-normalized median centered expression levels of Fgf13 across MMTV-PyMT tumors shows a significant reduction of expression in E2F1 KO tumors (p = 0.002, SAM: q = 0.01, 1.4 fold change). (**B**) Kaplan-Meier analysis of distant metastasis free survival across a dataset of human breast cancer^39^ shows that high expression of Fgf13 is associated with accelerated onset of metastatic progression (HR = 1.59, logrank p = 2e^−05^). (**C**) Kaplan-Meier analysis of distant metastasis free survival across a dataset of human breast cancer^39^ shows that high expression of Fgf13 is associated (but not statistically significant) with accelerated onset of metastatic progression in estrogen receptor (ER) positive breast cancers. (**D**) Kaplan-Meier analysis of distant metastasis free survival across a dataset of human breast cancer^39^ shows that high expression of Fgf13 is associated with accelerated onset of metastatic progression in ER-negative breast cancers (HR = 2.27, logrank p = 0.0075). (**E**) Kaplan-Meier analysis of distant metastasis free survival across a dataset of human breast cancer^39^ shows that high expression of Fgf13 is associated (but not statistically significant) with accelerated onset of metastatic progression in luminal A breast cancers. (**F**) Kaplan-Meier analysis of distant metastasis free survival across a dataset of human breast cancer^39^ shows that high expression of Fgf13 is associated with accelerated onset of metastatic progression in luminal B breast cancers (HR = 1.94, logrank p = 2e^−04^). (**G**) Kaplan-Meier analysis of distant metastasis free survival across a dataset of human breast cancer^39^ shows that high expression of Fgf13 is associated with accelerated onset of metastatic progression in basal-like breast cancers (HR = 1.89, logrank p = 0.034). (**H**) Kaplan-Meier analysis of distant metastasis free survival across a dataset of human breast cancer^39^ shows that high expression of Fgf13 is associated with accelerated onset of metastatic progression in Her2-enriched breast cancers (HR = 2.79, logrank p = 0.026).

Since we had identified a number of pathways and genes altered in E2F1^−/−^ tumors which were previously identified as metastasis driver genes, we sought to identify novel regulators of metastasis from the genes downregulated in E2F1^−/−^ tumors. Candidate genes for further testing were prioritized by selecting genes that had not previously been demonstrated to regulate breast cancer metastasis, that were E2F target genes, and correlated with decreased time to distant metastasis across several human breast cancer human breast cancer subtypes. With this pipeline we identified differential expression of fibroblast growth factor 13 (*Fgf13*). As shown in Fig. 3A, expression was significantly reduced 1.4-fold in E2F1^−/−^ tumors (q = 0.01, p = 0.02). Analysis of the 500 bp sequence upstream of the transcriptional start site (TSS) revealed seven E2F binding motifs in human and ten in the mouse sequence for this gene. High expression of Fgf13 was significantly associated with faster onset of distant metastasis across all cases of breast cancer (Fig. 3B) but was not significant across all Er+ tumors (Fig. 3C). However, it was significantly associated with accelerated time to distant metastasis in ER- tumors (Fig. 3D). Observing intrinsic subtypes, Fgf13 was not associated with metastasis of Luminal A tumors (Fig. 3E). However, in Luminal B, Basal-like, and Her-2 enriched tumors high expression Fgf 13 was predictive of early onset metastasis (Fig. 3F–H respectively).

**Figure 4:**
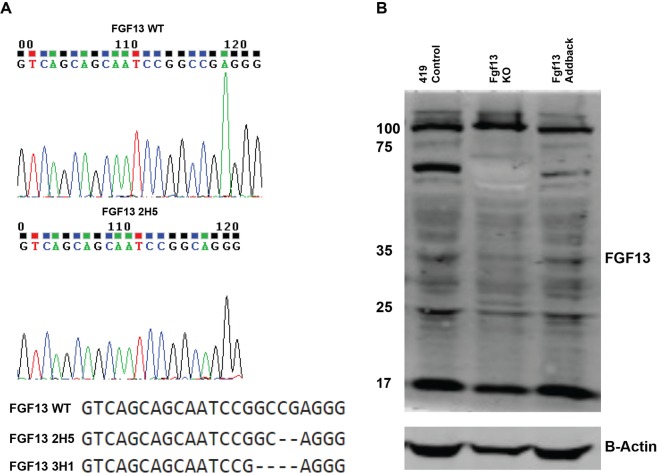
Sequence trace and alignment for CRISPR-mediated Fgf13 knockout. (**A**) Sequence trace for Fgf13 WT cells and Fgf 13 knockout clone 2H5. (**B**) Western blot analysis for Fgf13 WT cells, Fgf13 knockout cells, and Fgf13 add back cells.

To test this gene for metastatic behavior, we utilized a PyMT-derived cell line (PyMT 419 cells)^53^ and a CRISPR (clustered regularly interspaced short palindromic repeats) approach to create Fgf13 knockout cells. Figure 4A shows an example of sequence trace for Fgf13 control and a knockout lines. Western blot analysis confirmed that protein levels of Fgf13 were impacted by CRISPR gene editing (Fig. 4B). In addition, we transduced our knockout lines with a vector to restore Fgf13 and confirmed re-addition by Fgf13 by western blot (Fig. 4B).

**Figure 5:**
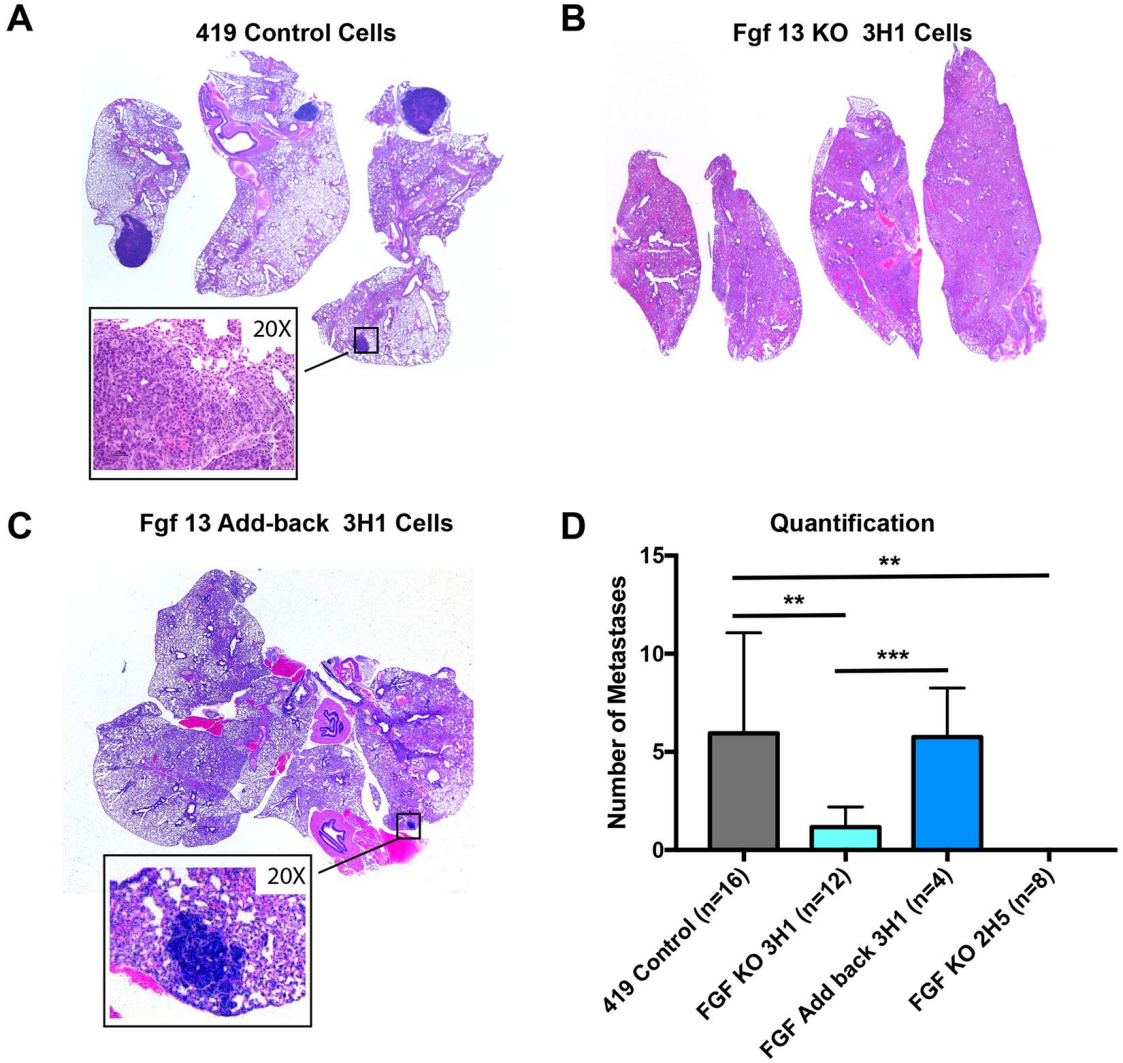
Fgf13 function in metastatic colonization of the lungs. (**A**)Representative image of lungs from mice receiving 419 control cells via a tail vein injection shows robust metastatic colonization of the lungs. (**B**) Lungs of mouse receiving the Fgf13 knockout clone 3H1 shows that loss of Fgf13 impaired tumor cell colonization of the lungs. (**C**) Lungs of mouse receiving the Fgf13 addback (to clone 3H1) shows that restoration of Fgf13 allows tumor cell colonization of the lungs. Quantification of FGF13 KO and addback clones compared to MMTV-PyMT 419 cell line control cells (black bar) shows a significant reduction in metastasis to the lungs in mice receiving or FGF13 (green bars) knockout cells (unpaired t-test, p < 0.05; each clone compared by to wild type control).

We opted to assess colonization capacity by tail vein injection. In this setting 50,000 cells were injected into the tail vein and lung colonization was examined 21 days later. In mice receiving control cells, robust metastatic colonization of the lungs was observed (Fig. 5A). In addition, metastatic tumors were found sporadically throughout the mouse at other sites. Loss of Fgf13 dramatically limited the ability of the tumor cells to form colonies at the lung (Fig. 5B) and re-expression of Fgf13 restored colonization ability (Fig. 5C). There was a significant reduction in the number of lesions present in the lungs for mice receiving Fgf13^−/−^ cells (Fig. 5D). Together, these results show that Fgf13 regulates breast cancer pulmonary colonization, a critical step in metastasis.

### Investigating Fgf13 function

**Figure 6:**
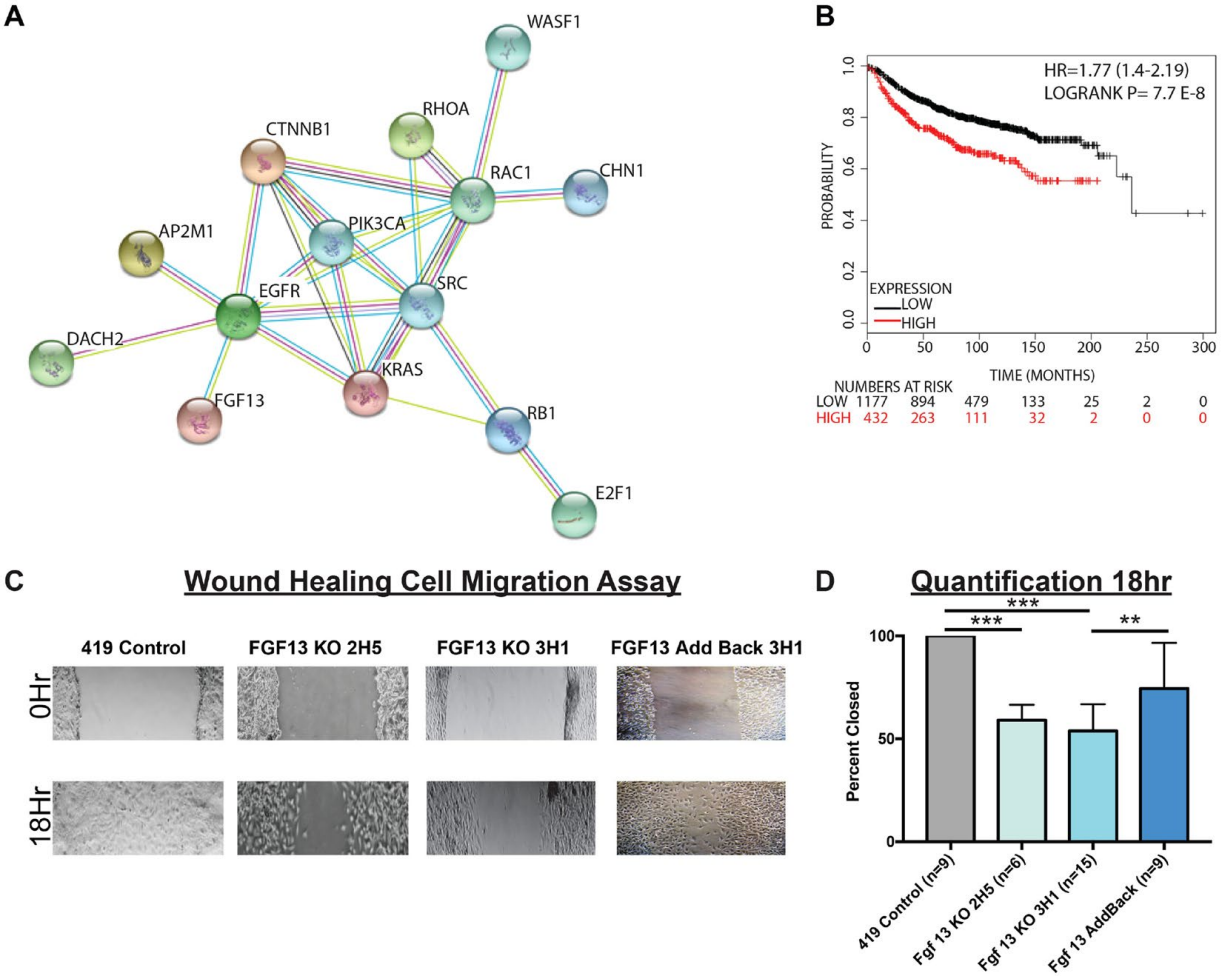
WGCNA analysis of MMTV-PyMT tumors predicts Fgf13 function in cell motility with confirmation by wound healing assay. (**A**) String interaction network for Fgf 13 covariance network genes and cell signaling pathways with low activity in E2F1^−/−^ tumors reveals an association for genes with a gene significance score greater than 0.60 shows FGF13 many of the the FGF13 metagene are demonstrated interactors, including genes that function in cell motility (Rac1, Wasf1, Chn1). In addition, these covariance network genes associate with the Ras, Egfr, RhoA, Src, Rb, E2F1, and beta-catenin pathways. (**B**) Kaplan Meier analysis for the Fgf 13 covariance network genes as a signature shows these genes are significantly associated with earlier human breast cancer metastasis. (**C**) Scratch assay photos showing impaired migratory ability of 419 control cells, FGF13 KO cells, and Fgf13 add-back cells. (**D**) Quantification wound closure at 18 hours in of 419 control cells, FGF13 KO cells, and Fgf13 add-back cells.

To predict Fgf13 function, we examined the Fgf13 covariance network using WGCNA analysis of primary MMTV-PyMT E2F wild type tumors (Supplemental File 2), identifying 38 probes corresponding to 35 genes that tightly correlate with Fgf13 expression (gs threshold = 0.6, p-value < 0.0005). MSigDB revealed a significant association with a signature for genes up-regulated in invasive ductal carcinoma relative to ductal carcinoma *in situ* (Supplemental File 3, p-value = 1.02 e^−4^, FDR q-value = 3.97 e^−2^). We were also interested to note association with the Rac1 cell motility pathway (Supplemental File 2, p-value = 5.38 e^−7^, FDR q-value = 1.29 e^−3^). Key to this association was strong covariance with Rac1 (gs = 0.60), the GTPase activating protein chimerin 1 (Chn1, gs = 0.60), and Wasf1 (which acts downstream of Rac1 to regulate the cytoskeleton, gs = 0.61). Matching this association, Fgf13 was part of the KEGG pathway for regulation of the actin cytoskeleton. Testing these alterations for the presence of an interaction network with Rb-E2F1 and the pathways altered in E2F1^−/−^ tumors illustrated a relationship between Fgf13, cytoskeleton and motility genes, and pathways predicted to have low activity in E2F1^−/−^ tumors (Fig. 6A). WGCNA identified additional genes with cytoskeleton regulatory function such Tubulin beta 6 (Tubb6) and microtubule-associated protein 1B (Mtap1B). In addition, given Fgf13’s published role in neuronal cell differentiation^54^, we were interested to note this as another prominent theme amongst the Fgf13 metagene (Table 1). In testing these genes as a signature for association with human breast cancer metastasis events, we found that high expression of these genes was significantly associated with a shorter time to distant metastasis across breast cancer tumors (Fig. 6B).

**Table 1:** Developmental And Migration Themes In Fgf13 Metagene.

Gene	Gene Signicance Score	Function/Expression Pattern	Ref
BEX4	0.72	Family of Proteins involved in Neuronal Differentiation.	^74^
KCDN2	0.67	Potassium ion channel protein expressed in neurons	^75^
Mtap1b	0.67	Stabilizes microtubules and implicated in neuronal migration	^76^
KLF16	0.64	Regulation of neurite outgrowth	^77^
SEMA3C	0.61	Regulation of neuronal migration	^78^
WASF1	0.61	Neurite outgrowth	^79^
Rims2	0.6	Important to neuron synapse function	^80^
Itm2a	0.6	Motor neuron marker	^81^
Rac1	0.6	Neuronal cell migration	^82^
Chn1	0.6	Regulation of neuron dendrite morphology	^83^

To test for a possible function in cell motility, we assessed the migratory capacity of the MMTV-PyMT control cells and Fgf13 KO cells using a scratch assay (Fig. 6C). While control cells were able to close the scratch at 18 hours, the Fgf13 KO clones demonstrated a significant defect in cell migration. Re-expression of Fgf13 restored migratory capacity of these cells, with wounds nearly closed by 18 hours (Fig. 6D). Together, these data confirm the bioinformatic predictions that Fgf13 functions in cell migration and provides a likely explanation for the colonization defects associated with Fgf13 loss.

## Discussion

Using a transcriptomic approach, we investigated the mechanism by which E2Fs regulate breast cancer metastasis^11^. Here we used bioinformatic analysis to compare global gene expression differences between E2F^WT/WT^ MMTV-PyMT tumors and E2F1^−/−^ MMTV-PyMT tumors. We demonstrated that loss of E2F1 led to decreased activity in several key signaling pathways previously demonstrated to regulate metastasis (Fig. 2). Many of the genes downregulated in E2F1^−/−^ tumors correlate with a faster progression to metastatic disease in human clinical data were significantly associated with the gene expression response to hypoxia (Supplemental File 1).

The majority of the genes associated with hypoxia were also direct E2F1 target genes. Hypoxia has been described as a master regulator of metastasis due to the result of gene expression changes brought about by hypoxia response^37^. These gene expression changes enable tumor cells to progress through multiple checkpoints during the metastatic cascade. This includes promoting angiogenesis, epithelial to mesenchymal transition, tumor cell invasion, remodeling of the extra cellular matrix, and increasing cell migration^37,55–57^. Hypoxia also upregulates genes which facilitate tumor cell intravasation, survival in the blood stream, extravasation and colonization at distant organs^37^. Consistent with processes associated with hypoxia response, we previously observed angiogenesis defects and a decrease in circulating tumor cells suggesting intravasation defects or an inability for metastatic cells to resist anoikis^11^. Our prior work had shown that these vascular defects were associated with a significant reduction in the major angiogenesis signaling molecule, Vegfa. This study expands on this finding and adds to the growing body of research that has demonstrated E2F1 as a key regulator of angiogenesis^18,58–60^; suggesting E2F1 regulates many genes within the hypoxia response program to potentially coordinate the development of tumor vasculature.

In addition to hypoxia response, E2F1^−/−^ tumors had significantly lower expression of genes previously associated with metastasis. *In vitro* studies have demonstrated that Areg^47^, Tead1^48^, Coro1C^49^, Lama5^50^, Tgm2^51^ and Fgf 7^52^ are involved in cell migration and invasion features of tumor cells. The reduced expression of these genes involved in invasion phenotypes may provide additional mechanistic information to explain our previous finding that E2F1^−/−^ tumors had possible invasion/intravasation problems indicated by a reduction in circulating tumor cells^11^.

The data presented here also demonstrated a role for Fgf13 in colonization of the distal site in metastasis. Fgf13 is a nonsecretory protein of the FGF family^54^ and we show that loss of Fgf13 in tumor cells blocked colonization of the lungs in a tail vein injection setting. In order to investigate the mechanism for this block of metastatic capacity, we identified that genes that co-vary with Fgf13 using WGCNA. Amongst these genes, we noted several genes associated with cell motility were in an interaction network with FGF13. This led us to predict that FGF13 may function in cell migration. We confirmed this prediction using a scratch assay where both Fgf13 knockout clones demonstrated impaired cell migration. Interestingly, the role of Fgf13 in neuron development also highlights its role in cell migration while detailing a mechanism of Fgf13 induced microtubule stabilization^54^. Within the Fgf13 covariate network, we also observe many genes consistent with microtubule stabilization and neuron development (Table 1). This agreement between the two studies might suggest conserved mechanisms for cell migration in tumor cells and in neurons. Further, drawing from our study and that of Wu *et al*.^54^ predicts that the possible function of Fgf13 in metastasis may be attributed to a function in cell migration via microtubule stabilization.

As a whole, this study shows that the metastatic defects associated with E2F1 loss could be due to an inability to properly initiate a gene expression response to hypoxia. Importantly, many of the genes downregulated in E2F1^−/−^ tumors associated with hypoxia response have been shown as regulators breast cancer metastasis. We identified that the E2F1 target gene Fgf13 was critical for pulmonary colonization, potentially through a cell migration mechanism; possibly providing a means for E2F1 to regulate cell migration. Collectively, this study furthers our initial characterization of E2F1’s regulation of metastasis by identifying the metastasis associated gene expression response to E2F1 loss.

## Methods

### RNA and microarray

Preparation of RNA samples from flash frozen tumors was done using the Qiagen RNeasy kit after roto-stator homogenization. RNA from 17 Myc induced tumors was submitted to the Michigan State University Genomics Core facility for gene expression analysis using Mouse 430A 2.0 Affymetrix arrays.

### Gene expression analysis

Raw intensity.CEL files were processed and RMA normalized using Affymetrix Expression Console. Gene expression data is deposited on the Gene Expression Omnibus under the accession number GSE104397. Unsupervised class discovery was done using Consensus Cluster Plus^23^. For class discovery, we mapped Affymetrix probes to their gene symbol using the platform table deposited on the Gene Expression Omnibus (GPL8321). Given the presence of multiple probes for single genes, we collapsed duplicate genes to their mean expression in each sample. This approach reduced our dataset of 22690 probes down to 12847 gene symbols. Next, we median centered the gene expression values across the samples in the dataset. Finally, we filtered genes using a requirement of a standard deviation greater than 0.5. This resulted in 1,303 genes used for class discovery and consensus clustering. We used 1,000 iterations and 90% item (sample) resampling to evaluate 2–10 potential clusters (groups/classes). Consensus cumulative distribution function (CDF) plots are also generated using Consensus Cluster Plus^23^. Silhouette width^24^ was used to assess validity of each cluster using the R package ‘Cluster’^61^. Pathway activation was predicted according to previous studies^14,32,62^. Single sample gene set enrichment analysis was done for Hallmark^36^ gene sets using the ssGSEAProjection module on Broad Institute’s Gene Pattern^63^. Significance analysis of microarrays^38^ was used to compare E2F ^WT/WT^ and E2F1^−/−^ tumors in a fold change analysis. Direct E2F1 target genes were identified using ChIP-base^40^ and data from a previous ChIP-seq experiment^22^. Kaplan-Meier plots were generated using using KMPLOT.com to query human breast cancer expression and clinical data^64^. Significant overlaps with previously established gene sets were detected using the molecular signatures database^36^. Interaction networks were assembled using www.string-db.org^65^. Weighted correlation network analysis was implemented according to published protocols^66^ and using a gene significance score threshold of 0.6 to select genes for further analysis.

### Cell culture

MMTV-PyMT 419 cells were a gift from Dr. Stuart Sell and Dr. Ian Guessand have been previously characterized^53^. All tumor cells were cultured in Dulbecco’s Modified Eagle’s Medium, 3.7 g/L of NaHCO3, 3.5 g/L d-glucose, 5 ug/mL insulin, 1 ug/mL hydrocortisone, 5 ng/mL Egf, 35 ug/mL BPE, 50 ug/mL gentamicin, 1X Antibiotic/Antimycotic, and 10% fetal bovine serum. Media was set to a pH of 7.4.

### CRISPR

Sequence for Fgf13 was obtained from the UCSC genome browser^67^. Guide sequences were predicted by submitting exon (using only those that were common across all Fgf13 isoforms) sequence using the CRISPR design tool at: http://crispr.mit.edu/. Oligos for guide sequence assembly were designed by adding a ‘G’ followed by ‘CACC’ at the 5′ end of the guide sequence. For the complementary DNA to the guide, add ‘CAAA’ to the 5′ end. Oligonucleotide sequences are as follows:

Fgf13 5′: CACCGTCAGCAGCAATCCGGCCGA

Fgf13 3′: AAACTCGGCCGGATTGCTGCTGACC

Oligonucleotides for guide sequence assembly were ordered from integrated DNA technologies https://www.idtdna.com/site. Oligos were diluted to a concentration of 100 uM in water. To anneal the oligonucleotides 5 uL of the forward and 5 uL of the reverse oligo are incubated in 10 uL of 2X annealing buffer (10 mM Tris, pH 7.5–8.0, 50 mM NaCl, 1 mM EDTA) at 95 degrees Celsius for 4 minutes, and then cooled to room temperature. The annealed oligonucleotides were inserted into the PX458 vector from Addgene (#48138). Confirmation of the cloned guide sequence was done using Sanger sequencing.

PyMT 419 cells were transfected according to a previously described protocol^68^. To select clones, GFP positive cells were sorted into 96 well plates using fluorescence activated cell sorting. Knockout clones were screened for by PCR amplifying a ~300 bp amplicon with the PAM sequence centrally located within the PCR product. PCR products were resolved on a 3% agarose gel, extracted with the QIAquick Gel Extraction Kit, and were submitted for sanger sequencing.

PCR Amplification Primers:

Fgf13 5′: 5′-TGTTCTAACTTCCAGAAAGGCATA-3′

Fgf13 3′: 5′-CAGTGGTTTGGGCAGAAAAT-3′

For sequencing, a nested primer with the following sequence was used.

Fgf13 5′: 5′-CACACCCATATAAGTATTGACTTTCA-3′

Knockout and add-back were confirmed by western blot according to published methods^28^. For western blots, we used a polyclonal Fgf13 antibody (Invitrogen PA527302) and for beta-actin we used a monoclonal antibody (Cell Signaling #4970). The Fgf13 ORF was purchased from GenScript in the pcDNA3.1 vector and were subsequently digested with EcoRI and XhoI cloned into the EcoRI and XhoI sites of pLXSN. Retrovirus was packaged in HEK-293GPG as previously described (90). Briefly, a confluent 10-cm plate of HEK-293GPG cells was transfected with 5 µg of pLXSN-mADM or pLXSN-mFGF13 and virus containing media was subsequently harvested. Subconfluent 10 cm plates (~20%confluence) of 419 cells were infected with 1 mL of non-concentrated viral supernatant and infected cells were selected with 300 µg/mL G418.

### *In vitro* Assays

To measure cell migration, wound healing assays were performed in the presence of 2 ug/mL Mitomycin C using standard methods^69^. Photomicrographs were taken at 0 hour and 18 hours.

### *In vivo* assays

Animal protocols used for this study were approved by Michigan State University IACUC Committee and conducted according to national and institutional guidelines. All mice were in the FVB background. For tail vein injection, MMTV-Cre control mice were used to avoid immune response to the middle T antigen^70–72^. For control cells and each knockout clone, 50,000 cells were injected into the bloodstream via the tail vein. After 21 days, mice were euthanized. Lungs were resected for H&E staining to detect metastases. To calculate significance, unpaired T-tests were done in graphpad prism.

## Supplementary information

Supplementary Information

## References

[1] Program, S. R. SEER 18 2004-2010, All Races, Females by SEER Summary Stage 2000. *National Cancer Institute* (2011).

[2] Bos PD (2009). Genes that mediate breast cancer metastasis to the brain. Nature.

[3] Kang Y (2003). A multigenic program mediating breast cancer metastasis to bone. Cancer Cell.

[4] Minn AJ (2005). Genes that mediate breast cancer metastasis to lung. Nature.

[5] Nguyen DX, Bos PD, Massague J (2009). Metastasis: from dissemination to organ-specific colonization. Nat Rev Cancer.

[6] Bidus MA (2006). Prediction of lymph node metastasis in patients with endometrioid endometrial cancer using expression microarray. Clinical Cancer Research.

[7] Van’t Veer LJ (2002). Gene expression profiling predicts clinical outcome of breast cancer. nature.

[8] Winnepenninckx V (2006). Gene expression profiling of primary cutaneous melanoma and clinical outcome. Journal of the National Cancer Institute.

[9] Andrechek ER (2015). HER2/Neu tumorigenesis and metastasis is regulated by E2F activator transcription factors. Oncogene.

[10] Chakrabarti R (2012). Elf5 inhibits epithelial mesenchymal transition in mammary gland development and breast cancer metastasis by transcriptionally repressing Snail2/Slug. Nature cell biology.

[11] Hollern DP, Honeysett J, Cardiff RD, Andrechek ER (2014). The E2F transcription factors regulate tumor development and metastasis in a mouse model of metastatic breast cancer. Molecular and cellular biology.

[12] Wang W (2007). Coordinated regulation of pathways for enhanced cell motility and chemotaxis is conserved in rat and mouse mammary tumors. Cancer research.

[13] Guy CT, Cardiff RD, Muller WJ (1992). Induction of mammary tumors by expression of polyomavirus middle T oncogene: a transgenic mouse model for metastatic disease. Mol Cell Biol.

[14] Hollern, D. P. & Andrechek, E. A genomic analysis of mouse models of breast cancer reveals molecular features of mouse models and relationships to human breast cancer. *Breast Cancer Research* 16 (2014). 10.1186/bcr3672 PMC4078930 25069779

[15] Attwooll C, Lazzerini Denchi E, Helin K (2004). The E2F family: specific functions and overlapping interests. EMBO J.

[16] Dyson N (1998). The regulation of E2F by pRB-family proteins. Genes Dev.

[17] Leone G (2001). Myc requires distinct E2F activities to induce S phase and apoptosis. Mol Cell.

[18] Field SJ (1996). E2F-1 functions in mice to promote apoptosis and suppress proliferation. Cell.

[19] Murga M (2001). Mutation of E2F2 in mice causes enhanced T lymphocyte proliferation, leading to the development of autoimmunity. Immunity.

[20] Humbert PO (2000). E2f3 is critical for normal cellular proliferation. Genes Dev.

[21] To B, Andrechek ER (2018). Transcription factor compensation during mammary gland development in E2F knockout mice. PloS one.

[22] Bieda M, Xu X, Singer MA, Green R, Farnham PJ (2006). Unbiased location analysis of E2F1-binding sites suggests a widespread role for E2F1 in the human genome. Genome research.

[23] Wilkerson MD, Hayes DN (2010). ConsensusClusterPlus: a class discovery tool with confidence assessments and item tracking. Bioinformatics.

[24] Rousseeuw PJ (1987). Silhouettes: a graphical aid to the interpretation and validation of cluster analysis. Journal of computational and applied mathematics.

[25] Pfefferle AD (2013). Transcriptomic classification of genetically engineered mouse models of breast cancer identifies human subtype counterparts. Genome Biol.

[26] Zhao X, Rødland EA, Tibshirani R, Plevritis S (2015). Molecular subtyping for clinically defined breast cancer subgroups. Breast Cancer Research.

[27] Andrechek ER (2009). Genetic heterogeneity of Myc-induced mammary tumors reflecting diverse phenotypes including metastatic potential. Proceedings of the National Academy of Sciences.

[28] Hollern D, Yuwanita I, Andrechek E (2013). A mouse model with T58A mutations in Myc reduces the dependence on KRas mutations and has similarities to claudin-low human breast cancer. Oncogene.

[29] Carvalho CM (2008). High-dimensional sparse factor modeling: applications in gene expression genomics. Journal of the American Statistical Association.

[30] Hollern DP, Swiatnicki MR, Andrechek ER (2018). Histological subtypes of mouse mammary tumors reveal conserved relationships to human cancers. PLoS genetics.

[31] Fan C (2011). Building prognostic models for breast cancer patients using clinical variables and hundreds of gene expression signatures. BMC medical genomics.

[32] Bild AH (2006). Oncogenic pathway signatures in human cancers as a guide to targeted therapies. Nature.

[33] Yoshioka K, Nakamori S, Itoh K (1999). Overexpression of small GTP-binding protein RhoA promotes invasion of tumor cells. Cancer research.

[34] Myoui A (2003). C-SRC tyrosine kinase activity is associated with tumor colonization in bone and lung in an animal model of human breast cancer metastasis. Cancer Research.

[35] Zhang D (2009). Epidermal growth factor receptor tyrosine kinase inhibitor reverses mesenchymal to epithelial phenotype and inhibits metastasis in inflammatory breast cancer. Clinical Cancer Research.

[36] Liberzon A (2011). Molecular signatures database (MSigDB) 3.0. Bioinformatics.

[37] Lu X, Kang Y (2010). Hypoxia and hypoxia-inducible factors: master regulators of metastasis. Clinical cancer research.

[38] Tusher VG, Tibshirani R, Chu G (2001). Significance analysis of microarrays applied to the ionizing radiation response. Proc Natl Acad Sci USA.

[39] Györffy B (2010). An online survival analysis tool to rapidly assess the effect of 22,277 genes on breast cancer prognosis using microarray data of 1,809 patients. Breast cancer research and treatment.

[40] Yang J-H, Li J-H, Jiang S, Zhou H, Qu L-H (2013). ChIPBase: a database for decoding the transcriptional regulation of long non-coding RNA and microRNA genes from ChIP-Seq data. Nucleic acids research.

[41] Schoeffner DJ (2005). VEGF contributes to mammary tumor growth in transgenic mice through paracrine and autocrine mechanisms. Laboratory investigation.

[42] Zhou Z (2014). Autocrine HBEGF expression promotes breast cancer intravasation, metastasis and macrophage-independent invasion *in vivo*. Oncogene.

[43] Gibert B (2012). Targeting heat shock protein 27 (HspB1) interferes with bone metastasis and tumour formation *in vivo*. British journal of cancer.

[44] Taylor AP, Goldenberg DM (2007). Role of placenta growth factor in malignancy and evidence that an antagonistic PlGF/Flt-1 peptide inhibits the growth and metastasis of human breast cancer xenografts. Molecular cancer therapeutics.

[45] Zhang H (2012). HIF-1-dependent expression of angiopoietin-like 4 and L1CAM mediates vascular metastasis of hypoxic breast cancer cells to the lungs. Oncogene.

[46] Xing RH, Rabbani SA (1996). Overexpression of urokinase receptor in breast cancer cells results in increased tumor invasion, growth and metastasis. International journal of cancer.

[47] Higginbotham JN (2011). Amphiregulin exosomes increase cancer cell invasion. Current Biology.

[48] Zhang H (2009). TEAD transcription factors mediate the function of TAZ in cell growth and epithelial-mesenchymal transition. Journal of biological chemistry.

[49] Wang J (2014). miR-206 inhibits cell migration through direct targeting of the actin-binding protein Coronin 1C in triple-negative breast cancer. Molecular oncology.

[50] Giannelli G, Falk-Marzillier J, Schiraldi O, Stetler-Stevenson WG, Quaranta V (1997). Induction of cell migration by matrix metalloprotease-2 cleavage of laminin-5. Science.

[51] Mangala L, Fok J, Zorrilla-Calancha I, Verma A, Mehta K (2007). Tissue transglutaminase expression promotes cell attachment, invasion and survival in breast cancer cells. Oncogene.

[52] Zang XP, Pento JT (2000). Keratinocyte growth factor-induced motility of breast cancer cells. Clinical & experimental metastasis.

[53] Ma J (2012). Characterization of mammary cancer stem cells in the MMTV-PyMT mouse model. Tumor Biology.

[54] Wu Q-F (2012). Fibroblast growth factor 13 is a microtubule-stabilizing protein regulating neuronal polarization and migration. Cell.

[55] Fujiwara S (2007). Silencing hypoxia-inducible factor-1α inhibits cell migration and invasion under hypoxic environment in malignant gliomas. International journal of oncology.

[56] Krishnamachary B (2006). Hypoxia-inducible factor-1-dependent repression of E-cadherin in von Hippel-Lindau tumor suppressor–null renal cell carcinoma mediated by TCF3, ZFHX1A, and ZFHX1B. Cancer research.

[57] Steinbrech DS (1999). Fibroblast response to hypoxia: the relationship between angiogenesis and matrix regulation. Journal of surgical Research.

[58] Engelmann D (2013). E2F1 promotes angiogenesis through the VEGF-C/VEGFR-3 axis in a feedback loop for cooperative induction of PDGF-B. Journal of molecular cell biology.

[59] Fontemaggi G (2009). The execution of the transcriptional axis mutant p53, E2F1 and ID4 promotes tumor neo-angiogenesis. Nature structural & molecular biology.

[60] Qin G (2006). Cell cycle regulator E2F1 modulates angiogenesis via p53-dependent transcriptional control of VEGF. Proceedings of the National Academy of Sciences.

[61] Nguyen DX (2009). WNT/TCF signaling through LEF1 and HOXB9 mediates lung adenocarcinoma metastasis. Cell.

[62] Gatza ML (2010). A pathway-based classification of human breast cancer. Proc Natl Acad Sci USA.

[63] Reich M (2006). GenePattern 2.0. Nature genetics.

[64] Szász, A. M. *et al*. Cross-validation of survival associated biomarkers in gastric cancer using transcriptomic data of 1,065 patients. *Oncotarget* 49322–49333 (2016). 10.18632/oncotarget.10337 PMC5226511 27384994

[65] Franceschini A (2013). STRING v9. 1: protein-protein interaction networks, with increased coverage and integration. Nucleic acids research.

[66] Langfelder P, Horvath S (2008). WGCNA: an R package for weighted correlation network analysis. BMC bioinformatics.

[67] Kent WJ (2002). The human genome browser at UCSC. Genome research.

[68] Liang X (2015). Rapid and highly efficient mammalian cell engineering via Cas9 protein transfection. Journal of biotechnology.

[69] Selvaraj N, Budka JA, Ferris MW, Jerde TJ, Hollenhorst PC (2014). Prostate cancer ETS rearrangements switch a cell migration gene expression program from RAS/ERK to PI3K/AKT regulation. Mol Cancer.

[70] Fluck, M. M. & Schaffhausen, B. S. Lessons in signaling and tumorigenesis from polyomavirus middle T antigen. *Microbiol Mol Biol Rev*; **73**: 542–563, Table of Contents (2009) 10.1128/MMBR.00009-09 PMC2738132 19721090

[71] Lukacher AE (1995). Susceptibility to tumors induced by polyoma virus is conferred by an endogenous mouse mammary tumor virus superantigen. The Journal of experimental medicine.

[72] Qiu TH (2004). Global Expression Profiling Identifies Signatures of Tumor Virulence in MMTV-PyMT-Transgenic Mice Correlation to Human Disease. Cancer research.

[73] Subramanian A (2005). Gene set enrichment analysis: a knowledge-based approach for interpreting genome-wide expression profiles. Proceedings of the National Academy of Sciences of the United States of America.

[74] Calvo L (2015). Bex3 dimerization regulates NGF-dependent neuronal survival and differentiation by enhancing trkA gene transcription. Journal of Neuroscience.

[75] Meng-chin AL, Lee H, Kornblum HI, Nelson SF, Papazian DM (2014). Kcnd2 Mutation Associated with Autism and Epilepsy Impairs Inactivation Gating in Kv4. 2 K+ Channels. Biophysical Journal.

[76] Moon HM, Wynshaw-Boris A (2013). Cytoskeleton in action: lissencephaly, a neuronal migration disorder. Wiley Interdisciplinary Reviews: Developmental Biology.

[77] Wang J (2016). Novel Roles and Mechanism for Krüppel-like Factor 16 (KLF16) Regulation of Neurite Outgrowth and Ephrin Receptor A5 (EphA5) Expression in Retinal Ganglion Cells. Journal of Biological Chemistry.

[78] Wiegreffe C (2015). Bcl11a (Ctip1) controls migration of cortical projection neurons through regulation of Sema3c. Neuron.

[79] Dehmelt L, Poplawski G, Hwang E, Halpain S (2011). NeuriteQuant: an open source toolkit for high content screens of neuronal morphogenesis. BMC neuroscience.

[80] Kaeser PS, Deng L, Fan M, Südhof TC (2012). RIM genes differentially contribute to organizing presynaptic release sites. Proceedings of the National Academy of Sciences.

[81] Shoemaker LD, Arlotta P (2010). Untangling the cortex: Advances in understanding specification and differentiation of corticospinal motor neurons. Bioessays.

[82] Kawauchi T, Chihama K, Nabeshima Y, Hoshino M (2003). The *in vivo* roles of STEF/Tiam1, Rac1 and JNK in cortical neuronal migration. The EMBO Journal.

[83] Buttery P (2006). The diacylglycerol-binding protein α1-chimaerin regulates dendritic morphology. Proceedings of the National Academy of Sciences of the United States of America.

